# Quantitative analysis of epithelial cells in urine from men with and without urethritis: implications for studying epithelial: pathogen interactions *in vivo*

**DOI:** 10.1186/1756-0500-2-139

**Published:** 2009-07-16

**Authors:** Rebecca Wiggins, Patrick J Horner, Kate Whittington, Christopher H Holmes

**Affiliations:** 1 Department of Clinical Sciences South Bristol, Obstetrics and Gynaecology, University of Bristol, St Michael's Hospital, Southwell St, Bristol, UK; 2 The Milne Centre Genito-Urinary Clinic, Bristol Royal Infirmary, Lower Maudlin St, Bristol, UK; 3 Laboratories of Integrative Neuroscience and Endocrinology, University of Bristol, Dorothy Hodgkin Building, Whitson St, Bristol, UK; 4 Department of Medical Biochemistry and Immunology, Cardiff University, The Tenovus Building, Academic Avenue, Heath Park, Cardiff, UK

## Abstract

**Background:**

Epithelial cells in first catch urine (FCU) specimens from 87 men with and without urethritis were quantified. Epithelial cells were broadly categorised into transitional and squamous populations using morphological characteristics and immunostaining with anti-pan leukocyte and anti-cytokeratin monoclonal antibodies.

**Findings:**

The majority (77/87 = 89%) of samples contained both transitional (76/87 = 87%; range 1 × 10^4 ^– 6 × 10^5^, median 6 × 10^4^) and squamous (57/87 = 66%; range 1 × 10^4 ^– 8 × 10^5^, median 2 × 10^4^) epithelial cells. The number of transitional cells correlated with the number of squamous cells (Spearman's rho = 0.697 p < 0.001). Squamous, but not transitional, cell numbers correlated with leukocyte numbers (Spearman's rho = 0.216 p = 0.045 and rho = 0.171 and p = 0.113, respectively). However there was no significant difference in epithelial cell numbers between men with and without urethritis. Nevertheless, some men with urethritis had relatively high numbers of transitional cells in their FCU. Transitional cells were morphologically heterogeneous and appeared to display complex cytokeratin phenotypes.

**Conclusion:**

Further studies are required to explore the complexity of epithelial cell populations in urine. These would provide novel opportunities for studying cellular interactions of *C. trachomatis *in male urethral infections, about which little is currently known.

## Findings

We have undertaken a preliminary quantitative analysis of epithelial cells in the urine of men with urethritis and compared the cellular profiles to those without urethritis. We wished to test whether the presence of epithelial cells has any significance in relation to urethritis and *C. trachomatis *and *N. gonorrhoeae *infection. Epithelial cells and leukocytes, identified using appropriately specific monoclonal antibodies, were quantified in first catch urine (FCU) specimens from men with and without acute urethritis.

To our knowledge, there has been no systematic attempt to quantify epithelial cells in FCU specimens, or examine their identity. Similarly, the significance for disease of epithelial cells in FCU and, indeed, whether there is a "normal" and "abnormal" epithelial cell content or profile in urine, is currently unknown.

Clinical guidelines for the diagnosis of urethritis rely solely on the presence of polymorphonuclear leukocytes (PMNLs). The incidence of other cells in urine does not form part of the current diagnostic criteria. Epithelial cells are frequently observed in urine and could originate from several sites within the male urinary and reproductive tracts including the kidney, bladder, prostate and urethra [1]. *Chlamydia trachomatis *and *Neisseria gonorrhoea *infect the genital tract mucosal epithelium, resulting in the production of pro-inflammatory cytokines [2,3]. The mucosal epithelium comprises a complex transitional epithelium with morphologies ranging from simple cuboidal/columnar to pseudo-stratified squamous [4,5]. The cells display different cytokeratin profiles, depending on their origin [4].

## Materials and methods

### Patients

Eighty -seven men attending an emergency walk-in genito-urinary medicine clinic were studied as previously described [6]. Fifty (57%) had urethritis and 37 (43%) did not. Urethritis was diagnosed and the FCU specimens processed as previously described [6]. *C. trachomatis *was detected in 17 men while 12 men had *N. gonorrhoeae*.

### Immunocytochemistry

Immunocytochemistry was performed to identify leukocytes and epithelial cells. Antibody F10-89-4 (European Collection of Animal Cell Cultures, Porton Down, Wiltshire, UK) against CD45 expressed by all leukocyte populations was used in the form of undiluted tissue culture supernatant. The pan-cytokeratin antibody cocktail AE1/AE3 (Serotec, Kidlington, Oxford, UK) was used at a 1:10 dilution in Tris-buffered saline (TBS). Antibody Ks 8.60 (Abcam, Cambridge, UK) identifying cytokeratins 1, 10, and 11 was used at a dilution of 1:100 in TBS.

Immunoperoxidase staining was carried out as described by Holmes *et al*. [7]. Briefly, 20 ml of urine from individual FCU specimens were pelleted by centrifugation at 400 × g for 10 minutes and the supernatant discarded. Cell pellets were re-suspended in 1 ml of Cytolyt preservative (Cytyc Ltd., West Sussex, UK), and stored overnight at 4°C. Cells were deposited on poly-L-lysine-coated microscope slides using a cytospin centrifuge. Slides were air-dried for one hour, incubated for a further hour with primary antibodies, washed twice in TBS, pH 7.4, and incubated for 30 minutes in rabbit anti-mouse immunoglobulins (DakoCytomation Ltd, Ely, Cambridgeshire) diluted 1:40 in TBS containing 10% normal human serum. After two washes in TBS, slides were developed using Sigma Fast diaminobenzidene tetrahydrochloride/H_2_O_2 _(Sigma Chemical Co., Dorset), counterstained with haemalum, progressively dehydrated in graded alcohols, cleared, and mounted. For immunofluorescence staining, slides were incubated in tetramethyl rhodamine isothiocyanate (TRITC) secondary antibody (Dako) diluted 1:20. After incubation for 30 minutes, the slides were washed in TBS and incubated with 4', 6-diamidino-2-phenylindole (DAPI) (Sigma) diluted 1:1000 to display nuclear staining. Slides were mounted with anti-fade reagent. Slides incubated in TBS in place of the primary antibodies were included in all tests as negative controls. Cells were counted in a Neubauer Haemocytometer in a 5 × 5 grid using a 40× objective; the minimum number of cells counted in each sample was 10, 000 [6].

### Statistical analysis

Total leukocytes and epithelial cells in each sample were calculated and values adjusted to represent a standardised 40 ml volume as described previously [6]. Statistical analyses were carried out using SPSS (SPSS *vs *14.0, SPSS inc., Chicago, USA). Mann-Whitney *U *and Spearman's rho non-parametric tests were used for comparisons.

## Results

### Immunostaining

**Figure 1:**
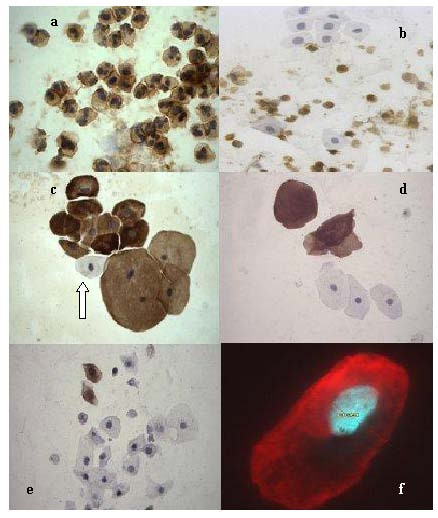
**Immunostaining of cell populations in FCU samples**. **a**. Anti-CD45 staining of a FCU specimen containing predominantly leukocytes, most of which are PMNLs (immunoperoxidase, × 100). **b**. Anti-CD45 staining of a FCU specimen containing predominantly CD45-negative epithelial cells (immunoperoxidase, × 40). **c**. Anti-pan cytokeratin staining of an epithelial cell cluster: one cell (arrowed) displays little or no staining (immunoperoxidase, × 40). **d**. Anti-cytokeratin 1, 10, 11 staining of mature squamous epithelial cells (immunoperoxidase, × 40). **e**. Anti-cytokeratin 1, 10, 11 staining of transitional type epithelial cells (immunoperoxidase, × 40). **f**. Anti-pan cytokeratin staining of an epithelial cell displaying a columnar type morphology (immunofluorescence, × 140).

**Figure 2:**
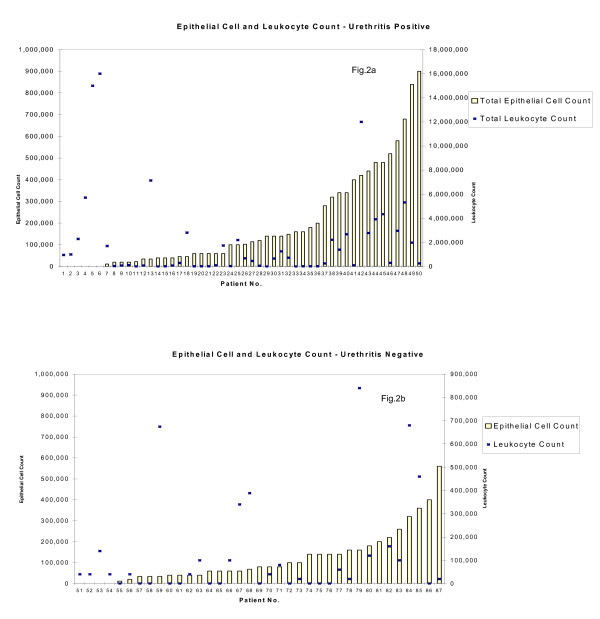
**a&b: Epithelial count (ranked) and leukocyte count (by patient sample) in urethritis positive (a) and urethritis negative (b) FCU specimens**.

Morphological characteristics and anti-CD45 and anti-cytokeratin reactivities of leukocytes and epithelial cells detected in the FCU specimens are illustrated in Fig. 1. Quantitative analysis of these populations is presented in Fig. 2. Figure 1a shows a FCU specimen in which the overwhelmingly predominant cell type was CD45-positive leukocytes, the majority of these being PMNLs [6]. While the majority of FCU specimens were found to contain at least some epithelial cells (see Fig. 2a &2b below), there was a wide variation in the proportion of CD45-positive leukocytes and CD45-negative epithelial cells. For example, the specimen illustrated in Fig. 1b, as well as containing CD45-positive leukocytes, also contained substantial numbers of CD45-negative epithelial cells. These latter cells exhibited a characteristic transitional morphology, displaying a typically round shape with a centrally disposed, round nucleus and high nuclear to cytoplasmic ratio (Fig. 1b).

Epithelial cell morphologies observed in these specimens are further illustrated in Fig. 1c–f where the cells were stained with anti-cytokeratin antibodies. Fig. 1c and Fig. 1f show staining with the pan-cytokeratin antibody cocktail AE1/AE3. Figure 1d and Fig. 1e are stained with an antibody specific for cytokeratins 1, 10, and 11. The latter reagent was used because it has previously been reported that cytokeratins 1, 10, and 11 are expressed only in squamous epithelium of the distal urethra in tissue sections [4]. We therefore reasoned that the antibody may detect cells from this location among the different epithelial populations in urine.

As expected, the pan-cytokeratin antibody displayed extensive reactivity with epithelial cells. Figure 1c shows the antibody stained transitional cells (cluster at top left) and also much larger cells having a centrally disposed, small, pyknotic nucleus typical of mature squamous epithelial cells (cluster at bottom right). Several preparations contained occasional cells having an apparent transitional morphology but showing little or no reactivity with the pan-cytokeratin antibody (arrow in Fig. 1c). It has recently been reported that the AE1/AE3 reagent used as a pan-cytokeratin antibody in this study does not detect all cytokeratins, including cytokeratins 17 and 18 (Antibody AE1/AE3 product information, Serotec). These AE1/AE3-negative presumptive epithelial cells may therefore express only very low levels of cytokeratins or, alternatively, may preferentially express only cytokeratins 17 and 18.

The anti-cytokeratin 1, 10, 11 antibody also showed strong reactivity with mature squamous epithelial cells but, in contrast to AE1/AE3, was generally unreactive with transitional cells (Fig. 1d). This is consistent with the reported expression of these cytokeratins only in squamous epithelium of the distal urethra. However, a more complex pattern was also observed in some FCU specimens using this reagent suggesting these cytokeratins are also expressed at other sites within the male urethra. The specimen shown in Fig. 1e contains some transitional cells that were clearly reactive with this antibody (Fig. 1e, top left). These antibody-positive transitional cells do not appear to display the relatively uniform, typically rounded morphology of transitional cells illustrated in Fig. 1b. Transitional cells clearly exhibit diverse morphologies, including the columnar-type appearance illustrated in Fig. 1f.

### Quantification of cells in FCU specimens

Total numbers of leukocytes and epithelial cells in the 87 FCU specimens are shown in Figure 2, where samples have been classified according to whether they are urethritis positive (Fig. 2a) or urethritis negative (Fig. 2b). To avoid bias, cell counts were undertaken without knowledge of their urethritis status, which was assigned only following cell quantification. Note that because leukocyte counts in some samples were very high (for example, sample number 6), Fig. 2a and Fig. 2b, showing the actual numbers detected, are presented on different scales (0 – 10^6 ^and 0 – 10^7^, respectively). Numbers of leukocytes and epithelial cells in these samples varied independently (Spearman's rho = 0.146, p = 0.18). In 63/87 (72.4%) samples, epithelial cells were in a relative minority compared with leukocytes. In the remaining 24 (27.5%) samples, epithelial cells either predominated over leukocytes or the two populations were present in similar numbers.

**Figure 3:**
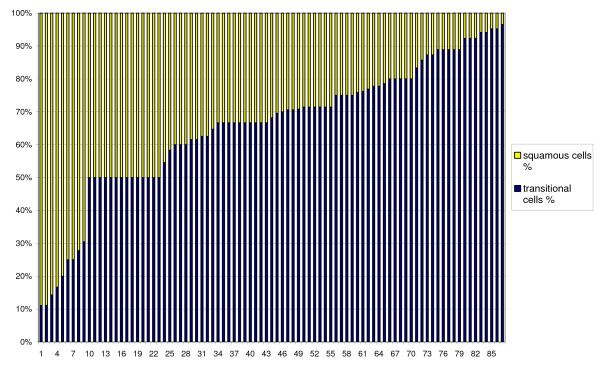
**Relative numbers of squamous and transitional cells in FCU specimens from men attending an emergency genito-urinary clinic (N = 87)**.

Epithelial cells with a transitional morphology can originate from several sites in the male urinary tract. Squamous cells are more likely to arise from the distal portion of the urethra, the *fossa navicularis*, which is mainly composed of thick, non-stratified squamous epithelium [4]. On the basis of their morphological characteristics, we quantified transitional and squamous cell populations within the overall epithelial cell population. Figure 3 is a graphical representation of the relative proportions of squamous and transitional cells in each sample. Transitional cells were detected in 87% (76/87) of samples (range 1 × 10^4 ^– 6 × 10^5^, median 6 × 10^4^) and squamous cells in 66% (57/87) of samples (range 1 × 10^4 ^– 8 × 10^5^, median 2 × 10^4^). Overall however, transitional cells were the predominant cell type in 64 of the 87 samples. The number of transitional cells correlated with the number of squamous epithelial cells (Spearman's rho = 0.697 p < 0.001). Squamous, but not transitional, cell numbers also correlated with leukocyte numbers (Spearman's rho = 0.216 p = 0.045 and rho = 0.171 and p = 0.113, respectively).

### Association of leukocyte and epithelial cell counts with urethritis

**Figure 4:**
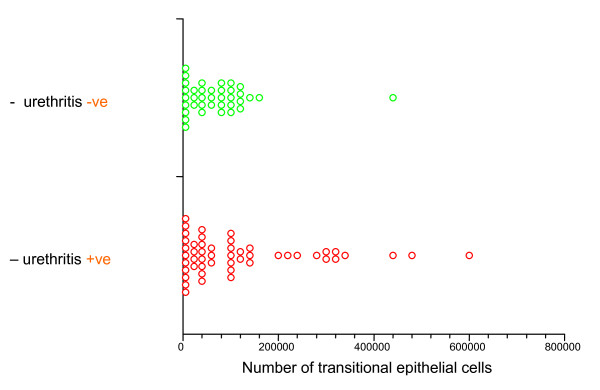
**Total transitional epithelial cell counts in FCU from men with and without urethritis (N = 87)**.

We examined the association of urethritis with the epithelial cell population in these FCU samples. There was no significant difference between total epithelial cell counts, squamous or transitional cell counts in men with and without urethritis (p = 0.39, p = 0.9 and p = 0.2, respectively, Mann-Whitney *U*). Figure 4 shows transitional cell counts in men with and without urethritis.

We also investigated whether there were differences in epithelial cell counts in FCU samples from men known to be infected with *N. gonorrhoea *and/or *C. trachomatis*. There were no significant differences in total epithelial, transitional or squamous epithelial cell counts between samples from men with *Chlamydia trachomatis *and *N. gonorrhoea *and men without these infections (p > 0.2). Similarly, in men with urethritis, there were no significant differences in the epithelial cell counts between men with either gonococcal or chlamydial infection and those without these infections (p > 0.2).

### Association between high leukocyte count and low epithelial cell count

We investigated the possibility of an inverse relationship between leukocyte and epithelial cell counts. There was no significant association, inverse or otherwise, between individuals with a high leukocyte count and a low epithelial cell count (Fisher's exact test, p = 0.255: data not shown).

## Discussion

Our quantitative study of cells in male FCU samples has identified the range of cells present in male urine based on a broad categorisation of epithelial cells into squamous and transitional cell morphologies. This offers a first step to investigate the range and incidence of different cell types *in vivo *and their relation to disease. Although squamous cells were correlated with the number of leukocytes, we did not find an association between the number of epithelial cells and urethritis or the presence of *C. trachomatis *or *N. gonorrhoeae*.

This was a small study which only distinguished between squamous and transitional cell populations. As the number of samples is limited, caution needs to be exercised in the interpretation of the results as type 2 statistical error cannot be excluded.

As we have previously shown for leukocytes [6], there is a wide variation in the total number of epithelial cells between different FCU specimens. Our study found that only squamous epithelial cells correlated with leukocyte numbers. Transitional epithelial cell counts were not associated with the detection of *C. trachomatis *or *N. gonorrhoeae *but this may be due to the small sample size. Nevertheless, the study indicates that high numbers of transitional epithelial cells will be present in some men with chlamydial urethritis (Figure 4).

## Conclusion

We have shown that epithelial cells are present in the majority of male FCU specimens and that this is a complex population. There was no significant association between total epithelial cell number, urethritis and detection *C. trachomatis *or *N. gonorrhoeae*. However, some men with urethritis have relatively high numbers of transitional epithelial cells in their FCU specimens. The squamous cell population also correlates with the presence of leukocytes. Further studies are required to explore the evident complexity of epithelial cell populations in urine.

## Competing interests

The authors declare that they have no competing interests.

## Authors' contributions

RW carried out the laboratory work and wrote the first draft of the text; PJH was one of the instigators of the project and contributed to the acquisition of patient samples and to the manuscript; KW was consulted about the project and contributed to the manuscript; and CHH was one of the instigators of the project, oversaw the laboratory work and contributed to the manuscript.
